# Long-Term Dietary Changes and Mortality among Participants of a Whole Food Plant-Based Pilot Study for Metastatic Breast Cancer

**DOI:** 10.1016/j.cdnut.2026.109405

**Published:** 2026-06-20

**Authors:** Thomas M Campbell, Erin K Campbell, Lisa M Blanchard, Nellie Wixom, Kevin M Spath, Luke J Peppone

**Affiliations:** 1 Department of Family Medicine, University of Rochester Medical Center, Rochester, NY, United States; 2 Department of Public Health Sciences, University of Rochester Medical Center, Rochester, NY, United States; 3 Clinical Research Center, University of Rochester Medical Center, Rochester, NY, United States; 4 Department of Surgery, Cancer Control, University of Rochester Medical Center, Rochester, NY, United States

**Keywords:** diet, nutrition, breast cancer, plant-based diet, vegan diet

## Abstract

**Background:**

There are limited data on dietary interventions among women with metastatic breast cancer. We previously conducted a feasibility trial of a whole food, plant-based (WFPB) diet in 32 women with metastatic breast cancer randomly divided 2:1 to WFPB (*n* = 21) or control (*n* = 11). The 8-wk intervention provided meals and weekly visits and resulted in beneficial changes but was not designed to support long-term behavior change.

**Objective:**

Given impressive short-term benefits, we wanted to know whether any dietary changes persisted even in the absence of support.

**Methods:**

We attempted to contact and re-consent survivors for a descriptive follow-up consisting of a 3-d food diary and quality-of-life questionnaires; medical records were reviewed to ascertain survival status. Two participants (1 control, 1 intervention) were withdrawn shortly after randomization in the pilot and were not included in this follow-up.

**Results:**

Overall, 2 of 20 (10%) participants originally assigned to the intervention and 5 of 10 (50%) assigned to control had died by the time of chart review. Among surviving participants, 11 of 18 intervention and 2 of 5 control participants completed the follow-up assessment, which occurred, on average, 5 y after their last study visit. Among intervention participants who completed dietary follow-up, mean energy intake remained lower. Although the intake of most food groups returned toward baseline, intake of refined grains and added fats remained lower at follow-up.

**Conclusions:**

In this small, exploratory, long-term follow-up, research participants maintained selective dietary changes 5 y after completing an 8-wk intervention. Although mortality differences require cautious interpretation, these descriptive findings suggest that longer, adequately powered studies are warranted.

This trial was registered at clinicaltrials.gov as NCT03045289 on 7 February 2017.

## Introduction

Historically, epidemiological studies associate mortality from breast cancer with a Western diet, rich in meat, fat, sugar, and processed foods [1,2]. Interrelated risk factors, including obesity, physical inactivity, and alcohol consumption are also known to increase risk [3]. Some experimental animal studies have suggested that specific foods or nutrients such as milk [4,5] and fat [6] increase breast cancer progression, but observational cohort studies have not consistently supported these findings [7,8], creating uncertainty regarding nutrition’s relationship to breast cancer risk. Fueling this uncertainty is the relative paucity of human dietary intervention trials with a duration long enough to link diet to cancer outcomes, particularly in women with advanced breast cancer. Most dietary interventions have been conducted in women with early-stage breast cancer who have completed their primary cancer treatment [9]. Furthermore, the largest and most well known of these intervention studies have arguably implemented modest dietary changes in the context of a rich, Western diet, as evidenced by limited or negligible weight loss among study participants assigned to dietary change [[10], [11], [12]].

Given the limited research related to *1*) implementing larger dietary changes and *2*) nutrition in subjects with more advanced breast cancer, we conducted an 8-wk pilot study to test the feasibility and preliminary effects of implementing a strict whole food, plant-based (WFPB) diet among women with metastatic breast cancer, all of whom were on stable systemic cancer therapy. We provided prepared meals and education related to dietary intervention for 8 wk and then study participation and contact with the study team ended. We found that recruitment and retention with this intervention was feasible and preliminary effects demonstrated weight loss, improved metabolic health, favorable changes related to biomarkers related to hormones, growth factors, and inflammatory markers, reduced serum amino acids, and significantly improved quality of life, particularly perceived cognitive function. These results have been previously published [[13], [14], [15], [16]]. The intervention was well liked and highly recommended among study participants.

Given this promising but brief study experience, we conducted long-term follow-up to describe current diet patterns, quality of life, and survival status among participants.

## Methods

This study was performed in compliance with recognized ethical guidelines, including the United States Common Rule. The study was reviewed and approved by the Research Subjects Review Board at the University of Rochester Medical Center. Written informed consent was obtained from all participants, and the trial was registered at clinicaltrials.gov (NCT03045289) on 7 February 2017. Methods for the pilot study have been described previously [16]. In brief, women with metastatic breast cancer were recruited between February 2018 and March 2022. Women were included if they had stable treatment regimens, as defined by no changes in cancer therapy in the past 6 wk and no anticipated treatment changes over the next month. They were randomly divided 2:1 to a WFPB intervention or control. The ad libitum WFPB diet included fruits, vegetables, legumes, whole grains, and potatoes and excluded all meat, including fish and seafood, dairy, added oils, and minimized added sugars and refined grains. Three prepared meals a day and snacks were provided. Participants could eat their own food instead of, or in addition to, provided meals, provided it was adherent to the dietary pattern. Intervention subjects attended weekly office visits, in person or by video conference, to review challenges, progress, and discuss an educational topic related to the diet. The pilot intervention was not designed to support long-term behavior change. It provided no support to participants as they learned to shop or cook for themselves and contact ended after their 8-wk participation was complete. No dietary pattern was mandated going forward and participants were encouraged to continue the study’s dietary changes only as much as they wished.

During the 8-wk study, dietary assessments included two 3-d food records (2 weekdays and 1 weekend day at baseline and week 8) and 3 unscheduled 24-h recalls. Unscheduled 24-h food recalls were conducted by phone by a dietitian (NW) at ∼2, 4, and 6 wk. Three-day food records and 24-h recalls were analyzed using Nutrition Data System for Research (NDSR), version 2017 (Nutrition Coordinating Center, University of Minnesota). A total of 94.3% of calories consumed by intervention participants were on plan, indicating that participants were highly adherent to the dietary pattern while enrolled.

Quality of life questionnaires were administered at baseline and 8 wk. Validated questionnaires included the Brief Fatigue Inventory (BFI), Functional Assessment of Cancer Therapy—Breast (FACT-B), and Functional Assessment of Cancer Therapy—Cognitive Function (FACT-Cog). Participants completed a demographic questionnaire at baseline and a feedback questionnaire at 8 wk.

In this current follow-up assessment presented here, we reviewed electronic medical records and attempted to recontact all participants beginning in October 2024 by sending 1 letter via postal service and making ≤3 phone calls. If we did not have current contact information or did not receive a response, we considered a participant “lost to follow-up.” We re-consented patients to conduct this follow-up assessment, which consisted of keeping a 3-d food record over 2 weekdays and 1 weekend day, completing the same quality of life surveys as they completed during their original study participation, and completing a brief survey regarding dietary habits. The food diary was reviewed and analyzed by a dietitian (NW) using NDSR, version 2021. Participants were given $50 gift cards for their participation in the follow-up assessment.

### Statistical methods

One participant assigned to the intervention group was withdrawn from the intervention within 1 wk of starting because of the pandemic shutdown. Because she had such limited exposure to the intervention, she was excluded from this analysis. Of the 11 intervention participants who completed food diaries at follow-up, 1 participant was excluded from dietary analysis because of outlier alcohol consumption on follow-up food record.

For food group serving analysis, normality tests indicated that the distribution of differences in food group consumption between timepoints was not normally distributed. Wilcoxon sign rank exact tests were performed to compare consumption of food groups at baseline with the final (8 wk) study assessment and from baseline to follow-up assessment, given that maintained changes from baseline to follow-up were the outcomes of interest. Similarly, Wilcoxon sign rank exact tests were performed to compare quality of life questionnaire scores from baseline to follow-up. Given the exploratory nature of these analyses and the small sample size, a conservative significance threshold of α = 0.05 was used, with a Benjamini–Hochberg (BH) false discovery rate correction applied to account for multiple comparisons.

## Results

**FIGURE 1:**
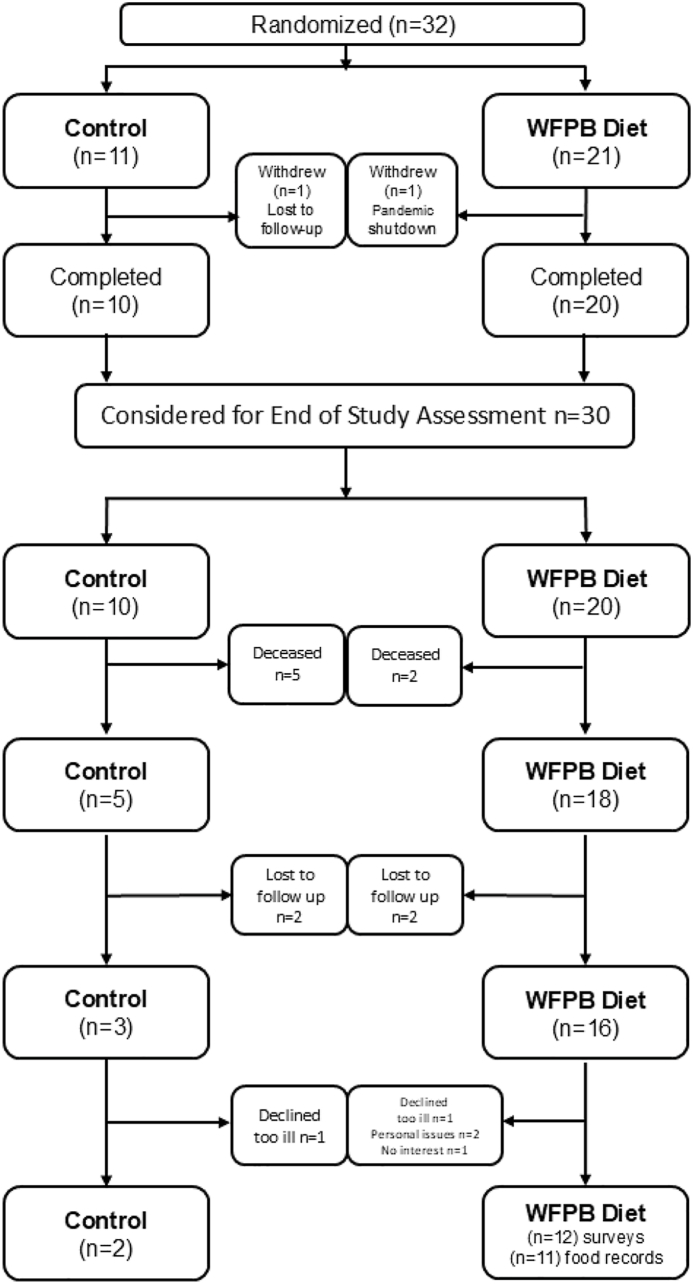
Consort diagram.

As shown in the consort diagram (Figure 1), 10% of the intervention group had passed away (2/20) and 50% of the control group had passed away (5/10). Of the 18 intervention participants still alive, 2 were lost to follow-up and 4 declined to participate, leaving 12 participants who completed follow-up assessment. Of those 12, 1 individual did not complete a food diary because of an illness in her family and extended travel and 1 individual was excluded from analysis because of high outlier alcoholic beverage serving consumption on follow-up. Of the 5 control participants still alive, 2 were lost to follow-up and 1 declined, leaving 2 participants who completed a food diary and questionnaires.

Table 1 shows the dietary intake, by food group servings, of 10 women in the intervention group with an evaluable food diary. There were large changes in most food group categories during the intervention, when meals were provided and weekly visits were held. The median daily intake from baseline to 8 wk increased in the following food group categories: fruits by 0.9 servings/d (*P =* 0.04, BH *P =* 0.06), vegetables by 5.8 servings/d (*P <* 0.01, BH *P* < 0.01), whole grains by 3.3 servings/d (*P <* 0.01, BH *P <* 0.01), nuts and seeds by 1 serving/d (*P <* 0.01, BH *P <* 0.01), legumes by 0.7 servings/d (*P =* 0.01, BH *P =* 0.02), meat alternatives by 0.6 servings/d (*P =* 0.08, BH *P =* 0.11), and nondairy milk by 0.3 servings/d (*P <* 0.01, BH *P <* 0.01). There were decreases in the following food group categories: refined grains by 4.4 servings/d (*P <* 0.01, BH *P <* 0.01), dairy by 1.1 servings/d (*P <* 0.01, BH *P =* 0.01), poultry by 1 serving/d (*P =* 0.03, BH *P =* 0.05), eggs by 0.1 serving/d (*P =* 0.02, BH *P =* 0.03), red and processed meats by 1.2 servings/d (*P <* 0.01, BH *P =* 0.02), and added fats by 4.5 servings/d (*P <* 0.01, BH *P =* 0.01).

**TABLE 1:** Median daily servings of foods consumed by intervention group participants with complete food records at all 3 timepoints (*n* = 10)^1^

Food group	Serving size units	Baseline diet	Week 8 WFPB diet	*P* value	BH-adjusted *P* value	5-y follow-up	*P* value	BH-adjusted *P* value
Fruits	½ cup cut fruit, 1 medium whole fruit, or ½ cup 100% juice	2.0 (0.6–2.6)	2.9 (1.4–4.0)	0.04	0.06	1.5 (0–3.0)	0.23	0.41
Vegetables	½ cup chopped vegetables, 1 cup raw leafy vegetables, or ½ cup tomato sauce	2.6 (1.5–3.6)	8.4 (5.0–10.9)	<0.01	<0.01	3.0 (0.4–3.7)	0.23	0.41
Grains								
Whole grains	½ cup cooked grain, hot cereal, or pasta, 16 g flour or cornmeal, 1 slice of bread or tortilla, or 1 ounce cold cereal or crackers	0.5 (0–0.8)	3.8 (2.7–6.8)	<0.01	<0.01	1.1 (0.5–1.6)	0.42	0.58
Some whole grains	Same grain serving sizes as above	0 (0–0)	0 (0–0.3)	0.63	0.70	0 (0–0)	0.75	0.90
Refined grains	Same grain serving sizes as above plus refined grain desserts (30 g cookie, 40 g brownie, 80 g cake, or 55 g donut)^2^	4.8 (3.8–7.0)	0.4 (0.2–1.1)	<0.01	<0.01	2.6 (1.6–3.2)	0.02	0.18
Dairy								
Dairy, full, reduced, and low-fat and fat-free	1 cup liquid milk or yogurt, 1 ½ ounces natural or 2 ounces processed cheese	1.1 (0.7–1.5)	0 (0–0)	<0.01	0.01	0.6 (0–1.2)	0.3	0.41
Frozen dairy desserts	½ cup ice cream or 85 g treat	0 (0–0)	0 (0–0)	1	1	0 (0–0.2)	1	1
Nuts, seeds, and legumes								
Nuts and seeds	½ ounce or 1 TB nut/seed butter	0.1 (0–0.9)	1.9 (0.6–2.6)	<0.01	<0.01	1.1 (0–2.3)	0.08	0.35
Legumes	½ cup cooked	0 (0–0.3)	0.7 (0.1–1.4)	0.01	0.02	0.2 (0–0.8)	0.56	0.72
Meat alternatives	1 ounce	0 (0–0)	0.6 (0–0.9)	0.08	0.11	0 (0–2.0)	0.38	0.56
Nondairy milks	1 cup	0 (0–0.2)	0.3 (0.2–0.4)	<0.01	<0.01	0.2 (0–0.6)	0.16	0.41
Poultry	1 ounce	1.0 (0–1.6)	0 (0–0)	0.03	0.05	0.8 (0–2.9)	1	1
Eggs	1 whole, 2 whites, or 2 yolks	0.1 (0–0.6)	0 (0–0)	0.02	0.03	0 (0–0.3)	0.22	0.41
Fish and shellfish	1 ounce	0 (0–1.8)	0 (0–0)	0.25	0.30	0 (0–0)	0.25	0.41
Red and processed meats	1 ounce	1.2 (0.2–2.1)	0 (0–0)	<0.01	0.02	0.4 (0–1.3)	0.20	0.41
Added fats	1 TS oil, 1 TS butter, margarine, or animal fat, 30 g dressing, 1 TB cream, or 1 ounce potato chips	4.8 (2.9–5.8)	0.3 (0–0.6)	<0.01	0.01	2.3 (1.6–3.2)	0.02	0.18
Added sugars	4 g sugar, ¼ cup syrup, 1 TB honey or jam, 40 g of candy	0.7 (0.1–2.7)	0.4 (0.2–1.0)	0.23	0.30	0.2 (0–0.5)	0.05	0.33
Sugar-sweetened beverages	8 ounces	0 (0–0)	0 (0–0)	1	1	0 (0–0)	1	1

Abbreviations: BH, Benjamini–Hochberg; TB, tablespoon; TS, teaspoon; WFPB, whole food, plant-based.

1 Diets were analyzed using NDSR. Values are medians (25th–75th percentile). Wilcoxon signed rank tests were used to compare food group servings of WFPB diet at 8 wk and follow-up diet with baseline diet.

2 There were no whole grain or some whole grain dessert servings eaten, only refined grain dessert servings.

After an average of 5 y after study participation, intake of many food groups had reverted toward baseline levels, but there remained some notable exceptions. Changes in food group consumption from baseline to long-term follow-up included decreases in refined grains by 2.2 serving/d (*P =* 0.02, BH *P =* 0.18) and added fats by 2.5 servings/d (*P =* 0.02, BH *P =* 0.18). There was a decrease in added sugar servings by 0.5 servings/d (*P =* 0.05, BH *P =* 0.33) and an increase in nuts and seeds by 1 serving/d (*P =* 0.08, BH *P =* 0.35) from baseline to follow-up.

Table 2 shows the changes in nutrient intake among the 10 women with evaluable food records at 5-y follow-up. At follow-up, energy consumption was reduced by 196 kcal/d (*P =* 0.03, BH *P =* 0.21) and fiber intake remained increased by 4.9 g/1000 kcal/d (*P =* 0.16, BH *P =* 0.43) but there were otherwise only small differences at follow-up in the intake of fat (% of kcal), carbohydrate (% of kcal), or protein (% of kcal) compared with baseline.

**TABLE 2:** Median daily nutrient intake of intervention group participants with complete food records at all three timepoints (*n* = 10)^1^

	Baseline diet	Week 8 WFPB diet	*P* value	BH-adjusted *P* value	5-y follow-up	*P* value	BH-adjusted *P* value
Energy (kcal)	1516.2 (1408.2–2124.8)	1347.4 (1109.2–1510.2)	<0.01	<0.01	1319.9 (1141.9–1601.9)	0.03	0.21
Fat (% of total kcal)	35.5 (30.8–38.4)	20.3 (16.9–25.2)	<0.01	<0.01	38.7 (34.5–40.2)	0.1	0.40
Saturated fat (% of total kcal)	11.4 (10.6–12.8)	3.7 (2.9–4.7)	<0.01	<0.01	14.4 (11.5–15.0)	0.32	0.43
Carbohydrate (% of total kcal)	48.8 (44.7–54.7)	68.6 (66.5–75.9)	<0.01	<0.01	47.2 (42.2–55.0)	0.28	0.43
Protein (% of total kcal)	15.6 (14.0–17.7)	14.6 (12.5–15.0)	0.09	0.09	16.7 (14.3–18.8)	0.23	0.43
% of total protein provided by plant sources	41.9 (36.7–51.0)	100.0 (99.7–100.0)	<0.01	<0.01	41.2 (31.4–81.5)	0.49	0.49
Dietary Cholesterol (mg)	161.2 (132.4–195.1)	0 (0–1.7)	<0.01	<0.01	181.9 (43.3–229.4)	0.49	0.49
Dietary fiber (g/1000 kcal)	9.5 (8.3–13.5)	31.0 (27.4–35.3)	<0.01	<0.01	14.4 (7.9–17.0)	0.16	0.43

Abbreviations: BH, Benjamini–Hochberg; WFPB, whole food, plant-based.

1 Values are medians (25th–75th percentile). Wilcoxon signed rank tests were used to compare energy and nutrient intake of WFPB diet at 8 wk and follow-up diet with baseline diet.

As shown in Table 3, reported quality of life was either unchanged or worse compared with baseline at long-term follow-up among the intervention respondents. Participant-reported cognitive function (FACT-Cog), which significantly improved during the 8-wk intervention, was statistically unchanged from baseline at long-term follow-up. Fatigue, as measured by the BFI, had worsened, both in terms of overall fatigue (*P =* 0.02, BH *P =* 0.14) and how much it interfered in daily life (*P =* 0.02, BH *P =* 0.14). The Social/Family Wellbeing subscale of the FACT-B survey was significantly worse at follow-up (*P =* 0.03, BH *P =* 0.14).

**TABLE 3:** Quality of life at baseline and long-term follow-up among intervention respondents^1^

	Better score	*n*^2^	Baseline	Long-term follow-up	*P* value	BH-adjusted *P* value
Functional Assessment of Cancer Therapy – Breast (FACT-B) – Overall	Higher	11	98.0 (90.5–108.0)	92.0 (84.3–106.0)	0.31	0.83
Breast Cancer Subscale	Higher	11	17.0 (16.0–22.5)	21.0 (15.0–22.5)	0.88	1
Functional Assessment of Cancer Therapy – General (FACT-G)	Higher	12	82.3 (71.3–89.5)	75.0 (66.9–83.1)	0.17	0.71
Functional Well Being	Higher	12	25.5 (22.5–29.5)	23.0 (19.8–27.3)	0.34	0.83
Physical Well Being	Higher	12	16.0 (13.8–17.0)	16.0 (11.5–19.3)	1	1
Emotional Well Being	Higher	12	11.5 (8.0–12.0)	11.5 (9.5–14.3)	0.96	1
Social/Family Well Being	Higher	12	28.5 (25.5–33.0)	24.5 (23.5–28.3)	0.03	0.14
Trial Outcome Index	Higher	11	63.0 (54.0–64.5)	58.0 (48.0–66.0)	0.62	0.96
Brief Fatigue Inventory – Overall	Lower	12	2.8 (1.9–3.8)	3.9 (2.8–5.8)	0.02	0.14
Fatigue Interference	Lower	12	2.2 (1.0–3.8)	3.8 (2.2–5.7)	0.02	0.14
Maximum Fatigue	Lower	12	5.5 (3.8–8.0)	6.5 (4–7.3)	0.59	0.96
Fatigue Severity	Lower	12	3.8 (2.7–6.7)	5.0 (3.8–6.0)	0.21	0.72
Functional Assessment of Cancer Therapy – Cognitive Function	Higher	12	102.8 (92.4–112.8)	99.2 (91.8–116.5)	0.91	1
Comments From Others	Higher	12	10.5 (9.8–12.0)	10.5 (9.0–11.3)	1	1
Perceived Cognitive Abilities	Higher	12	29.8 (28.4–30.4)	30.2 (29.3–31.2)	0.58	0.96
Perceived Cognitive Impairment	Higher	12	52.044.5–59.5)	47.0 (43.8–60.5)	0.53	0.96
Impact on Quality of Life	Higher	12	10.5 (8.0–12.8)	12.0 (6.8–13.0)	0.96	1

Abbreviation: BH, Benjamini–Hochberg.

1 Values are medians (25th–75th percentile).

2 One participant was excluded from some measures because of an unanswered question on baseline survey.

A brief 3-question survey was given to participants about dietary changes since the study. Table 4 shows a summary of the results from intervention respondents. 75% felt that their current diet included changes in the direction of a WFPB diet.

**TABLE 4:** Summary of feedback comments related to current diet compared with baseline diet among intervention respondents (*n* = 12)

To what extent did you continue the whole food, plant-based diet after the study intervention?	On a scale from 1–10 (10 being following it strictly), the mean response was 4.67 (SD 2.42)
Compared to your diet prior to the study, how is your current diet different (if at all)?	75% said they made positive changes aligned with WFPB: • Less meat/more legumes (33% of respondents) • More whole foods/whole grains, less processed foods (42% of respondents) • More plant-based (33% of respondents) • More fruits/veggies (33% of respondents) 25% said there was no difference between current diet and prestudy diet.
What has made it challenging to continue the whole food, plant-based diet?	Dislike of or inability to cook (33%) Not enough time (25%) Family won’t eat WFPB (25%) Other people and restaurants provide much of their food and it is not WFPB (25%) Difficulty getting the right foods (25%) Taste (17%) Sugar cravings (8%) Too restrictive (8%)

## Discussion

Women in our WFPB intervention group maintained select long-term dietary changes, still present an average of 5 y after their participation. They maintained decreases in energy intake, refined grains, added fats, and added sugar. These changes likely reflect less processed food intake, as reported by 42% of intervention respondents. The increases in nuts and seeds, legumes, plant-based milks, and calorie-adjusted dietary fiber compared with baseline suggest a modest retention of a “plant-forward” focus in some respondents’ intake, which was reported by 33% of respondents. Impressively, despite the original study not being designed to support long-term dietary change, 75% of intervention participants reported that they had made changes positively aligned with a WFPB diet while largely maintaining quality of life over 5 y. The intervention group had 10% mortality and the control group had 50% mortality, although this requires a cautious interpretation because of study limitations. Together, these findings further support the feasibility of a WFPB dietary intervention among women with advanced breast cancer.

The large difference in mortality in this follow-up analysis supports the need for further research but cannot be attributed to any single factor given the limited dietary adherence seen in this follow-up survey. There were many benefits seen during the 8-wk study that could plausibly affect cancer activity and progression, including improvements in metabolic health, hormonal markers, and intentional weight loss [15]. Angiogenic, inflammatory, serum amino acids, and other cancer-related markers improved within the intervention group at 8 wk [13,14]. These preliminary findings are consistent with the outcomes of a systematic review by Jiménez and Storz [17], which found that across a majority of the included randomized controlled trials and cohort studies, replacing animal foods or animal protein with plant foods or plant protein was associated with reduced cancer risk, including breast cancer risk.

There are important limitations to this follow-up analysis. There were no serum tests to examine whether any of the biomarker changes seen at 8 wk were maintained over time. Because there were no interim assessments, it is not possible to know how quickly dietary intake regressed over the poststudy time frame. It is possible that dietary intake regressed slowly and the biochemical changes seen in the pilot study persisted for years, which could plausibly impact cancer progression, but it is also possible the opposite is true. Calorie intake was reduced at 5 y among intervention respondents, suggesting lasting change, but the effect of decreased calorie intake alone is uncertain. Although reduced calorie intake may provide support for maintaining a healthier weight and improved cardiometabolic profile, some recommendations include increasing protein intake to preserve lean body mass [18], which is more difficult to do concurrent with reduced calorie intake.

Additionally, the small sample size limits our findings to an exploratory interpretation as a larger cohort would be needed to reinforce our conclusions statistically. The underpowered nature of our analyses limits our ability to confidently detect statistical significance, particularly after multiple comparison correction. Although some *P* values showed significance, those that did not remain significant after the BH corrections should be interpreted cautiously. The direction of changes in food group consumption is compelling, however, given the pattern of change. Decreased intake of red and processed meats, dairy, refined grains, added fats, and added sugars together with an increased intake of whole grains, nuts and seeds, legumes, and vegetables does not suggest random variation but instead a consistent pattern of changes in the direction of a WFPB dietary pattern, as reported by participants.

Approximately a third of the intervention group did not complete surveys or complete a food diary for this analysis. Assuming they did not retain any dietary changes from their study experience, it appears that roughly half of the intervention group did not retain changes to their diet (7 nonresponders plus 3 responders who reported that their current diet was no different from their baseline diet). Additionally, overall nutrient intakes suggest that residual changes from the baseline dietary pattern were limited in important ways, even among respondents. Given the short-term nature of the original study, the lack of design features that would support long-term behavior change, the extended length of time since participation, and the numerous barriers to incorporating large dietary changes faced by individuals with metastatic cancer, this was not surprising. Indeed, we find it impressive that as much as half of the group had retained some dietary changes, however limited.

Behavior change and maintenance is challenging. This has relevance beyond dietary change and impacts medication adherence as well. A 2012 systematic review of medication adherence studies in real-world settings found that up to two thirds of breast cancer survivors either discontinued early or did not adhere to adjuvant hormonal therapy for the recommended 5 y [19]. Adherence to chronic disease medications more generally has been found to be as low as 50%, with adherence rapidly dropping in the first year [20]. In some dietary studies, adherence sometimes starts to wane as intervention intensity decreases or the intervention stops altogether [12,21]. But other small randomized controlled trials in patients with multiple sclerosis [22], heart disease [23], or overweight/obesity [24] suggest that maintenance of large long-term dietary changes (1 y or more) is possible when utilizing strict WFPB interventions, even after primary interventions are complete.

What specifically matters, and how much, for more successful long-term adherence is up for debate. There are many interrelated factors inherent to dietary interventions that simultaneously vary across interventions. A 2018 report from the United States Preventive Services Task Force found intensive, multicomponent programs to have benefit for weight loss. Most programs they evaluated lasted 1–2 y, had 12+ meetings in the first year, and had a more intense “core” phase plus a maintenance phase [25]. A separate 2022 review of weight loss interventions found that to prevent weight regain an intervention duration of ≥12 wk is better, and sustained, regular contact, at last monthly, after the primary intervention, improves weight loss maintenance [26].

The most prevalent barriers reported by participants in our study included challenges around cooking or preferences of family/friends and/or restaurant food choices that were at odds with the dietary pattern. It takes weeks to months and multiple food exposures for tastes to change [27]. Our 8-wk pilot study supported large dietary changes during the intervention (94% of calories consumed were from on-plan foods), but future studies will require sufficient duration, design, and ongoing contact and support to foster long-term behavior change as participants tackle these challenges.

Despite participating in a study of just 8 wk duration, not designed to promote long-term dietary change, a significant portion of this cohort with metastatic breast cancer was able to carry forward some dietary changes for an average of 5 y after last study contact. The intervention group had lower mortality than the control group, although reasons for this are unknown. Together with the beneficial results seen at 8 wk of the original study, further research of this dietary pattern in this population is justified. Future research should be of greater duration and interventions should be designed to support long-term dietary change to better observe any effect on cancer outcomes. In addition, a study with a larger sample size would be warranted to further confirm these findings.

## Author contributions

The authors’ responsibilities were as follows – TMC: conceptualization, funding acquisition, investigation, methodology, project administration, resources, supervision, visualization, writing – original draft; EKC: conceptualization, data curation, formal analysis, investigation, methodology, project administration, supervision, visualization, writing – review and editing; LMB: conceptualization, data curation, formal analysis, investigation, project administration, visualization, writing – review and editing; NW: data curation, investigation, methodology, project administration, resources, writing – review and editing; KMS: data curation, formal analysis, writing – review and editing; LJP: conceptualization; writing – review and editing.

## Data availability

Data described in the manuscript, code book, and analytic code will be made available upon request pending reasonable request of the corresponding author.

## Declaration of Generative AI and AI-assisted technologies in the writing process

The authors declare that no generative AI or AI-assisted technologies were used in the writing of this manuscript.

## Funding

This work was supported by the Highland Hospital Foundation, which has received donations from The Ladybug Foundation, T. Colin Campbell Center for Nutrition Studies and multiple individuals. The funders had no role in study design; collection, analysis, and interpretation of data; or writing of the report and there were no restrictions regarding the submission of the report for publication.

## Conflict of interest

TMC: royalties from general interest books about plant-based nutrition (Benbella Books, Penguin Random House); EKC: conflicts of spouse (TMC); The rest of the authors declare no competing interests.
